# Utilizing data sampling techniques on algorithmic fairness for customer churn prediction with data imbalance problems

**DOI:** 10.12688/f1000research.72929.1

**Published:** 2021-09-30

**Authors:** Maw Maw, Su-Cheng Haw, Chin-Kuan Ho

**Affiliations:** 1 Faculty of Computing and Informatics, Multimedia University, Cyberjaya, Selangor, 63100, Malaysia

**Keywords:** Customer churn prediction, Data sampling techniques, Algorithmic fairness, Class imbalance problem

## Abstract

**Background:** Customer churn prediction (CCP) refers to detecting which customers are likely to cancel the services provided by a service provider, for example, internet services. The class imbalance problem (CIP) in machine learning occurs when there is a huge difference in the samples of positive class compared to the negative class. It is one of the major obstacles in CCP as it deteriorates performance in the classification process. Utilizing data sampling techniques (DSTs) helps to resolve the CIP to some extent.

**Methods:** In this paper, we review the effect of using DSTs on algorithmic fairness, i.e., to investigate whether the results pose any discrimination between male and female groups and compare the results before and after using DSTs. Three real-world datasets with unequal balancing rates were prepared and four ubiquitous DSTs were applied to them. Six popular classification techniques were utilized in the classification process. Both classifier’s performance and algorithmic fairness are evaluated with notable metrics.

**Results:** The results indicated that Random Forest classifier outperforms other classifiers in all three datasets and, using SMOTE and ADASYN techniques cause more discrimination in the female group. The rate of unintentional discrimination seems to be higher in the original data of extremely unbalanced datasets under the following classifiers: Logistics Regression, LightGBM, and XGBoost.

**Conclusions:** Algorithmic fairness has become a broadly studied area in recent years, yet there is a very little systematic study on the effect of using DSTs on algorithmic fairness. This study presents important findings to further the use of algorithmic fairness in CCP research.

## Introduction

Customer churn, the phenomenon in which customers are shifting to rival companies due to dissatisfaction with the existing services or to other inevitable reasons, ^1^ is one of the common issues usually encountered in every customer-oriented sector, including telecommunication. Customer churn prediction (CCP) is a supervised binary classification procedure that detects the potential churners before they are churned. Since there are no standardized principles for collecting data for CCP tasks, data distribution between classes will be varied from one data set to another. Therefore, one class might have extremely underrepresented compared to another class. In CCP, the target class is those being churned or not. To be exact, churn is always a minority class when the non-churn class usually comes in large numbers. Therefore, churn is used to consider a rare object ^2^ in service-based domains including telecom. Thus, telecom datasets always suffer from a class imbalance problem (CIP) and lead to a situation in which minority instances remain unlearned.

Advanced machine learning techniques can be applied to predict potential churners. Let us consider a dataset with 10,000 data instances with 10% of churn samples i.e., 1000 churners and 9,000 non-churners. Even if a carefully built model could predict 90% correctly on the minority class, it means 100 customers are misclassified to the wrong class. Suppose 60 churners are misclassified as non-churners, i.e., false negatives, the company will lose a huge amount of revenue since recruiting new customers is more expensive than keeping the existing ones. ^3^ Thus, the ultimate goal in the telecom sector is to increase profit by decreasing customer churn. Hence, CIP is a block when trying to achieve the major goal of CCP, since it degrades classification accuracy. Algorithmic fairness has become a very active research topic since ProPublica observed that the algorithms could yield discriminative outcomes, which impacted a minority group in real life. ^4^

Algorithmic fairness is monitored in line with the protected features or sensitive variables in the dataset. Sensitive data could generally be, but not limited to gender, race, age group or religion. Algorithmic fairness is achieved if the decisions generated by a model do not favor more or less any individual or a group. ^5^ The lesser the bias in the training data, the bigger the chance of achieving algorithmic fairness. However, it is almost not possible to train a zero-bias model since the historical data could have contained bias for many reasons. ^6^ The common reasons for bias in the training data involve the compounding of initial bias over time, using proxy variables, and unbalancing of sample size between minority and majority group. ^7^

In the CCP process, customers’ behavior is analyzed within specific time windows, for example within one month. ^8^ Once the prediction is done, the outcomes are reused as training data for the next prediction. Therefore, there are high chances to have repeated bias in the historical data without even noticing. One solution for CIP is to apply data sampling techniques (DSTs) to the training data. Since the major function of DSTs is to increase or decrease the sample instances to balance between majority and minority classes, there are changes in the number of samples in the different groups in the dataset. The main goal of this study is to explore and identify the impact of using DSTs on training data on algorithmic fairness in the CCP process. To the best of our knowledge, there is very little research concerning algorithmic fairness in the CCP process. We believe the findings of this study would provide valuable insights to future CCP research.

## Methods

Ethical Approval Number: EA1742021

Ethical Approval Body: Research Ethics Committee 2021, Multimedia University

**Figure 1.**
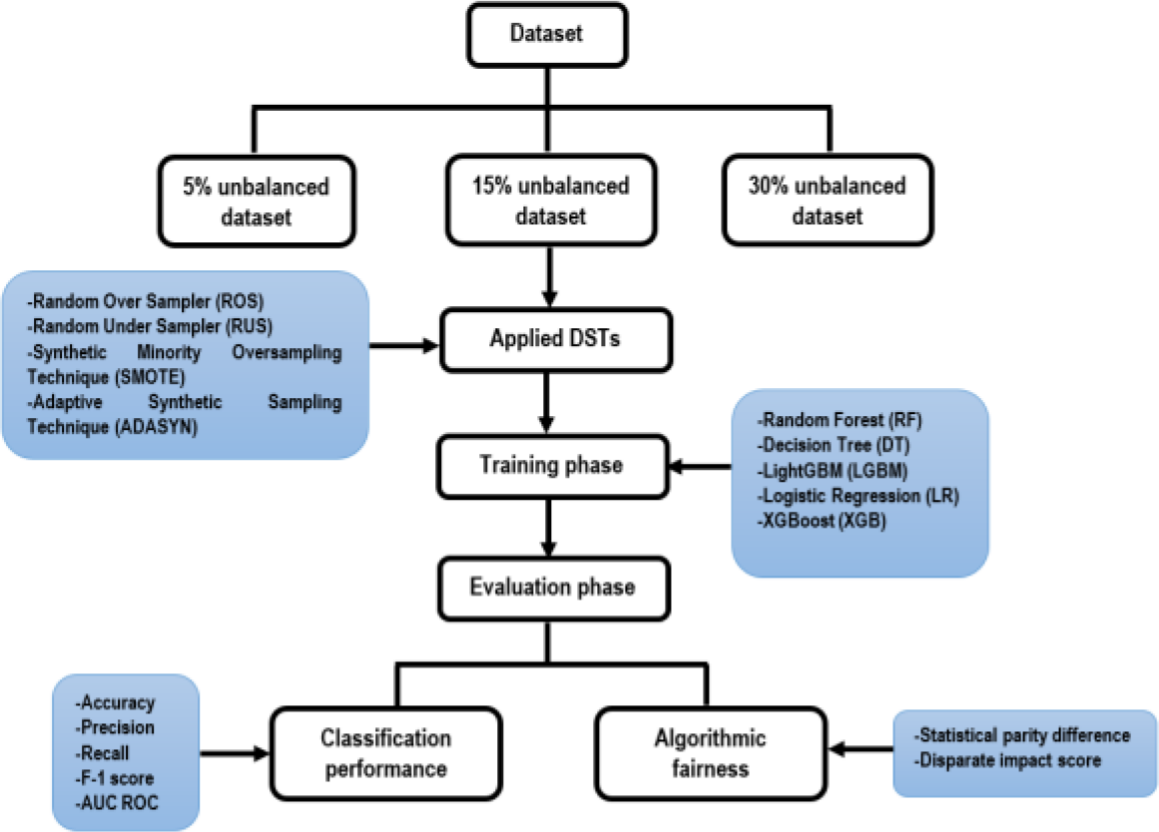
Procedures of the study.

In this study, the original data set is prepared to make three versions of unbalanced datasets, with rates of 5%, 15% and 30%. Each version is applied with four DSTs and compared the results with the unsampled original dataset to evaluate the classification performance and impacts on algorithmic fairness. The step-by-step methods to conduct the study are presented in Figure 1.

### Datasets

A real-world telecom dataset was provided by one of Malaysia’s leading telecom companies (see *Underlying data* for details on access to this dataset). The original dataset contains 1,265,535 customer records, which were collected from January 2011 to December 2011. Since the original data set is huge in volume, we randomly selected 100,000 records and utilized them for this study. We included demographics, call information, network usage, billing information, and customer satisfactory data in our dataset since they are considered as influential factors in the CCP process. ^9^^,^^10^ A total of 22 features were extracted after careful aggregation, i.e., new features were created based on the original data and some unnecessary features were deleted from it, and features are listed in Table 1.

**Table 1.** Features used in the real-world dataset.

No.	Name of the features	Description
1	Customer ID	Customer ID
2	Age	Age of customer
3	Is senior	Is the customer over 60 or not
4	Gender	Gender of customer
5	Is local	Is the customer a Malaysian or an international?
6	Race	Is the customer Malay or Indian or Chinese or Other?
7	Technical-problem-count	Total technical complaints and general complaints made by a customer
8	Complain-count	Total general complaints made by a customer
9	Avrg download	Average download rate
10	Avrg upload	Average upload rate
11	T-Location	The location where the customer registered for the service
12	HSBB area	Is the customer in the area where a high-speed connection is required or not
13	Speed	Broadband speed customer has registered for
14	Price start	The value of package customer has bought
15	Contract period	The contract period of the customer
16	Median- outstanding	Average overdue fees
17	Avrg local amt	Average amount spends for calling locally
18	Avrg std amt	Average amount spends for subscriber trunk dialing
19	Avrg idd amt	Average amount spends on international calls
20	Avrg voice usage	Average amount spends on voice calls
21	Avrg dialup amt	Average amount spends on dialup service
22	Churn	Whether the customer is churned or not

The final dataset was prepared with three different rates of unbalancing: 5%, 15%, and 30%. We created a Python script (see *Extended data*) which used the Pandas tool of Scikit-learn machine learning library to prepare three versions of datasets. We set up these specific rates because we wanted to experiment with extremely unbalanced cases up to intermediate levels.

### Data preprocessing

In the data preprocessing stage, we excluded any null values. Since we found only a few outliers in the selected dataset, we manually removed them without using any specific procedure. We applied four DSTs to the data: Random Over Sampler (ROS), Random Under Sampler (RUS), ^11^ Synthetic Minority Oversampling Technique (SMOTE), ^12^ and Adaptive Synthetic Oversampling Technique (ADASYN). ^13^ The selection of DSTs was based on their popularity and to know the impact of each of them on the algorithmic fairness in the CCP process.

### Classification of data

We applied six popular classifiers: Random Forest (RF), Decision Tree (DT), LightGBM (LGBM), Gradient Boosting (GB), Logistics Regression (LR), and XGBoost. ^14^ We created our own Python script (see *Extended* data) using Scikit-learn machine learning library to perform this step. After a careful exploratory data analysis, we dropped Customer ID, Avrg local amt, Avrg std amt, Avrg idd amt, Avrg dialup amt from the predictor variable list since they were weakly correlated to the target variable.

### Evaluation of experiment

We performed two evaluations: performance measures ^15^ and algorithmic fairness metrics. ^16^

***Performance measures***

In measuring the classifier's performance, we applied standard measures which are commonly used in most of machine learning classification tasks, including precision, recall and accuracy. We applied F-1 and AUC-ROC scores since accuracy alone is not enough to evaluate the actual performance of the classifiers. We created an own script (see *Extended data*) using Scikit-learn, a free machine learning software library for Python programming language. The performance of each classification was done as follows:

$$\text{Accuracy}=\frac{\mathrm{TP}+\mathrm{TN}}{\mathrm{TP}+\mathrm{TN}+\mathrm{FP}+\mathrm{FN}\;},$$

where

TP=true positive

TN=true negative

FP=false postive

FN=false negative

$$\text{Precision}=\frac{\text{True positive}}{\text{True positive}+\text{False positive}}$$

$$\text{Recall}=\frac{\text{True positive}}{\text{Ture positive}+\text{False negative}}$$

$$F1-\text{Score}=2*\frac{\text{Precision}*\text{Recall}}{\text{Precision}+\text{Recall}}$$

$$\mathrm{AUC}-\mathrm{ROC}=\frac{\sum \text{Rank}\;\left(+\right)-\left|+\right|*\left(\left|+\right|+1\right)/2}{\left|+\right|+\left|-\right|}\;\;\;\;\mathrm{where},\;\;\Sigma \;\mathrm{Rank}\;(+)\;\mathrm{is}\;\mathrm{the}\;\mathrm{sum}\;\mathrm{of}\;\mathrm{all}\;\mathrm{positive}\;\mathrm{classified}\;\mathrm{examples}\;|+|\;\mathrm{is}\;\mathrm{the}\;\mathrm{number}\;\mathrm{of}\;\mathrm{positive}\;\mathrm{examples}\;\mathrm{in}\;\mathrm{the}\;\mathrm{dataset}\;|-|\;\mathrm{is}\;\mathrm{the}\;\mathrm{number}\;\mathrm{of}\;\mathrm{negative}\;\mathrm{examples}\;\mathrm{in}\;\mathrm{the}\;\mathrm{dataset}$$

***Algorithmic fairness metrics***

We emphasized the assessment of whether the classifier is discriminated between women, a protected group, and men, a non-protected group. We applied two well-known fairness definitions in measuring algorithmic fairness, and utilized the popular AI-fairness 360 tool to calculate algorithmic fairness. ^16^

*Statistical parity (SP)*: Also known as an equal acceptance rate. SP is achieved if women have an equal probability to be predicted in the positive, i.e., churn class, as the men. ^17^

SP difference measures the difference of a specific outcome between the protected (female group) and non-protected (male group). The smaller the SP difference between the two groups, we can say that the model treats the unprotected group statistically similar to the protected group.

SP is calculated as follows:

$$\mathrm{Pr}\;\left(Y=1\right|\;\text{Group}=\text{male})=\mathrm{Pr}\;\left(Y=1\right|\;\text{Group}=\text{female}),\;\text{where}\;Y=\text{predicted decision}$$

*Disparate Impact (DI)*: Also known as indirect discrimination where no protected variables are directly applied, but biased outcomes are still produced relying on the variables correlated with protected variables. ^18^ The standardized threshold in calculation of DI is 0.8, which means the group whose DI values are under 0.8 are discriminated by the classifier.

The threshold value 80% is advised by the US Equal Employment Opportunity Commission. ^19^ The model could be DI-free when the value is larger than 80% but it should be lower than 125% according to. ^20^

DI is calculated as follows:

$$\mathrm{DI}=\frac{\mathrm{Pr}\left(Y=1\;\right|\text{Group}=\text{female})}{\mathrm{Pr}\left(Y=1\right|\text{Group}=\text{male})}\;\leq \;\tau =0.8,$$

where Y=predicted decision

## Results

The preliminary classification results for the datasets with different data unbalanced rates using four DSTs are shown in Tables 2– 4. Table 2 shows the specific results of classification performance gotten when testing on 5% of unbalanced rate with respect to the chosen classifiers and four DSTs.

**Table 2.** The classification results for the dataset with 5% unbalanced rate.

Classifier	5% imbalanced with ROS					5% imbalanced with RUS				
	Accuracy	Precision	Recall	F1-score	AUC-ROC	Accuracy	Precision	Recall	F1-score	AUC-ROC
RF	0.99	0.99	0.99	0.99	0.99	0.84	0.84	0.84	0.84	0.93
DT	0.98	0.98	0.98	0.98	0.97	0.80	0.80	0.80	0.80	0.79
LGBM	0.89	0.90	0.89	0.89	0.96	0.84	0.84	0.84	0.84	0.93
GB	0.85	0.85	0.85	0.85	0.99	0.85	0.85	0.85	0.85	0.93
LG	0.78	0.81	0.78	0.78	0.87	0.80	0.83	0.80	0.81	0.88
XGBoost	0.93	0.93	0.93	0.93	0.97	0.83	0.83	0.83	0.83	0.92

5% imbalanced with SMOTE					5% imbalanced with ADASYN				
Accuracy	Precision	Recall	F1-score	AUC-ROC	Accuracy	Precision	Recall	F1-score	AUC-ROC
0.98	0.98	0.98	0.98	0.99	0.98	0.98	0.98	0.98	0.99
0.96	0.96	0.96	0.96	0.96	0.96	0.96	0.96	0.96	0.96
0.98	0.98	0.98	0.98	0.99	0.98	0.98	0.98	0.98	0.99
0.96	0.96	0.96	0.96	0.99	0.96	0.96	0.96	0.96	0.99
0.79	0.81	0.79	0.79	0.87	0.77	0.81	0.77	0.77	0.84
0.98	0.98	0.98	0.98	0.99	0.98	0.98	0.98	0.98	0.99

**Table 3.** The classification results for the dataset with 15% unbalanced rate.

Classifier	15% imbalanced with ROS					15% imbalanced with RUS				
	Accuracy	Precision	Recall	F1-score	AUC-ROC	Accuracy	Precision	Recall	F1-score	AUC-ROC
RF	0.97	0.97	0.97	0.97	0.99	0.76	0.75	0.76	0.76	0.83
DT	0.93	0.93	0.93	0.93	0.93	0.68	0.68	0.68	0.68	0.68
LGBM	0.78	0.78	0.78	0.78	0.87	0.76	0.77	0.76	0.76	0.84
GB	0.76	0.77	0.76	0.76	0.84	0.76	0.76	0.76	0.76	0.84
LG	0.64	0.64	0.64	0.64	0.69	0.64	0.64	0.64	0.64	0.70
XGBoost	0.81	0.81	0.81	0.81	0.90	0.75	0.76	0.75	0.76	0.84

15% imbalanced with SMOTE					15% imbalanced with ADASYN				
Accuracy	Precision	Recall	F1-score	AUC-ROC	Accuracy	Precision	Recall	F1-score	AUC-ROC
0.93	0.94	0.93	0.93	0.97	0.93	0.93	0.93	0.93	0.97
0.89	0.89	0.89	0.89	0.89	0.88	0.89	0.88	0.89	0.88
0.94	0.94	0.94	0.94	0.97	0.94	0.94	0.94	0.94	0.97
0.91	0.92	0.91	0.91	0.96	0.91	0.92	0.91	0.91	0.96
0.64	0.64	0.64	0.64	0.70	0.59	0.59	0.59	0.59	0.63
0.94	0.76	0.94	0.94	0.97	0.94	0.94	0.94	0.94	0.97

**Table 4.** The classification results for the dataset with 30% unbalanced rate.

Classifier	30% imbalanced with ROS					30% imbalanced with RUS				
	Accuracy	Precision	Recall	F1-score	AUC-ROC	Accuracy	Precision	Recall	F1-score	AUC-ROC
RF	0.89	0.89	0.89	0.89	0.95	0.74	0.74	0.74	0.74	0.82
DT	0.83	0.83	0.83	0.83	0.83	0.66	0.66	0.66	0.66	0.66
LGBM	0.76	0.76	0.76	0.76	0.84	0.75	0.76	0.75	0.75	0.83
GB	0.74	0.75	0.74	0.74	0.82	0.75	0.76	0.75	0.75	0.82
LG	0.66	0.67	0.66	0.66	0.68	0.63	0.64	0.63	0.63	0.68
XGBoost	0.77	0.77	0.77	0.77	0.85	0.75	0.75	0.75	0.75	0.83

30% imbalanced with SMOTE					30% imbalanced with ADASYN				
Accuracy	Precision	Recall	F1-score	AUC-ROC	Accuracy	Precision	Recall	F1-score	AUC-ROC
0.86	o.86	0.86	0.86	0.92	0.86	0.86	0.86	0.86	0.92
0.79	0.79	0.79	0.79	0.79	0.79	0.79	0.79	0.79	0.79
0.86	0.87	0.86	0.86	0.92	0.86	0.87	0.86	0.86	0.93
0.84	0.86	0.84	0.84	0.91	0.84	0.85	0.84	0.84	0.91
0.66	0.67	0.66	0.66	0.68	0.55	0.92	0.55	0.65	0.63
0.86	0.86	0.86	0.86	0.92	0.86	0.87	0.86	0.86	0.93

Table 3 shows the details results of classification performance obtained when testing on 15% of unbalanced dataset with respect to the chosen classifiers and four DSTs.

Table 4 shows the details results of classification performance obtained when testing on 30% of unbalanced dataset with respect to the chosen classifiers and four DSTs.

In our study, we have observed that a variable, is-senior remained unbalanced even after applying the DSTs. The algorithmic fairness scores for each group with different unbalanced rates are described in Tables 5– 7. Table 5 shows the comparative results of SP difference and DI scores calculated on 5% unbalanced dataset and original dataset.

**Table 5.** The algorithmic fairness measures on 5% unbalanced dataset.

Algorithmic fairness metrics	5% original data	5% imbalanced with ROS	5% imbalanced with RUS	5% imbalanced with SMOTE	5% imbalanced with ADASYN
RF					
SP Difference	−0.0056	−0.0703	−0.0524	**0.1402**	**0.1401**
DI	0.80	0.86	0.89	**1.32**	**1.32**
LightGBM					
SP Difference	−0.0067	−0.0703	−0.0524	**0.1402**	**0.1390**
DI	**0.79**	0.86	0.89	**1.32**	**1.32**
GB					
SP Difference	−0.0057	−0.0854	−0.5456	**0.1349**	**0.1319**
DI	0.80	0.84	0.89	**1.31**	**1.29**
LR					
SP Difference	−0.0024	−0.0789	−0.0227	**0.1765**	**0.1591**
DI	**0.64**	0.94	0.96	**1.34**	**1.28**
XGBoost					
SP Difference	−0.0069	−0.0842	−0.5446	**0.1387**	**0.1383**
DI	**0.78**	0.85	0.89	**1.32**	**1.32**
DT					
SP Difference	−0.0073	−0.0698	−0.0397	**0.1331**	**0.1317**
DI	0.86	0.87	0.91	**1.30**	**1.29**

**Table 6.** The algorithmic fairness measures on 15% unbalanced dataset.

Algorithmic fairness metrics	15% original data	15% imbalanced with ROS	15% imbalanced with RUS
RF			
SP Difference	−0.009644	−0.058223	−0.049729
DI	0.871212	0.892587	0.891628
LightGBM			
SP Difference	−0.011044	−0.070341	−0.059666
DI	0.856964	0.853293	0.872208
Gradient Boosting			
SP Difference	−0.006168	−0.057517	−0.055826
DI	0.913621	0.874225	0.877351
Logistic Regression			
SP Difference	−0.007872	−0.1004	0.01865
DI	**0.711771**	**0.79581**	1.03868
XGBoost			
SP Difference	−0.011008	−0.069346	−0.059144
DI	0.864076	0.858386	0.874791
Decision Tree			
SP Difference	−0.016556	−0.05406	−0.016812
DI	0.900914	0.907414	0.967054

**Table 7.** The algorithmic fairness measures on 30% unbalanced dataset.

Algorithmic fairness metrics	30% original data	30% imbalanced with ROS	30% imbalanced with RUS
RF			
SP Difference	−0.035246	−0.046984	−0.043816
DI	0.824513	0.911002	0.905239
LightGBM			
SP Difference	−0.036931	−0.055603	−0.059621
DI	0.811701	0.882727	0.872917
Gradient Boosting			
SP Difference	−0.027146	−0.04126	−0.044747
DI	0.847531	0.910397	0.901757
Logistic Regression			
SP Difference	0.002424	0.011873	0.029787
DI	**1.042928**	**1.033943**	**1.063294**
XGBoost			
SP Difference	−0.036665	−0.056972	−0.058089
DI	0.826001	0.881622	0.877416
Decision Tree			
SP Difference	−0.28866	−0.033586	−0.028326
DI	0.911711	0.94218	0.944787

Table 6 displays the comparative results of SP difference and DI scores calculated on 15% unbalanced dataset and original dataset.

Table 7 describes the comparative results of SP difference and DI scores calculated on 30% unbalanced dataset and original dataset.

## Discussion

### Overview of experimental results

Recent works of algorithmic fairness research in machine learning applications is broadly organized into three main trends. Some studies emphasize enhancing or proposing better fairness notions and evaluation metrics in line with the domains concerned, ^17^^,^^21^ some focus on the ways to mitigate the bias in the classification process (which can further be divided into three main groups: pre-, in-, and post-processing techniques), ^22- 25^ while the last trend proposes how to maintain the ethical AI standards and policies in practicing machine learning applications in different sectors. ^26^^,^^27^

Despite some previous empirical studies on the impact of using preprocessing techniques on algorithmic fairness, the findings of previous works could not pinpoint the direct impact of using DSTs on algorithmic fairness. Lourenc and Antunes, ^28^ which is the closest work to our research, distinguish the effect of data preparation on algorithmic fairness. However, their work has been tested with two small datasets and provides general results of using random under- and over- DSTs. Importantly, their work fails to be tested on the widely-applied DSTs, SMOTE and ADASYN. In contrast, we apply real-world business data and show how different DSTs influence dissimilar levels of imbalance rate.

In the classification task, RF seems to be the best classifier since it yielded the best results over the other five models, while LR provided the worst scores for almost all metrics. It was observed that RUS worked better for the extremely unbalanced situation compared with 15% and 30% imbalanced rates. The best outcomes were found via ROS, SMOTE, and ADASYN in all different unbalanced rates, thus, could be concluded that oversampling techniques seem to provide more promising prediction results over undersampling techniques. This might be because the undersampling technique modifies the data by decreasing the majority of instances, which makes the dataset lack useful information for learning.

For all three unbalanced rates, the original dataset always gave less statistical parity differences (SPD) comparing to sampled datasets created using four DSTs, while datasets with RUS and ROS yield a slightly larger SPD but the statistics showed there is no disparate impact. However, we can hypothetically consider there might still be a bias as both RUS and ROS have their limitations. With RUS, important and essential data could have been removed and the classifier could provide a biased result since there was less information to learn from. On the other hand, with ROS, the prediction performance could also be biased due to the overfitting problem. In this sense, it is suggestible to apply different fairness measures and to compare the fairness scores. For the DI scores, if there is DI less than 0.8, there is indirect discrimination towards the unprotected group. The mathematical equivalence of DI suggests equalizing the outcomes between protected and unprotected groups. However, in reality, the conditions in the context of interest drive us to allow DI to a specific group up to some percentage. For example, in telecom CCP, the number of female customers could be very less than the dataset, since most males usually apply for a network plan representing the whole household. Therefore, we assume considering DI with 80% rule is reasonable.

In the 5% unbalanced original dataset, LGBM, LR and XG-Boost imposed with DI values of 0.79, 0.64, and 0.78 respectively. But there is no DI in the other two original datasets for 10% and 30%. This reveals that more discrimination could occur on a more unbalanced dataset. The analysis on all datasets with SMOTE and ADASYN provides alarming information on the classifier’s discrimination on the unprotected group. The 30% unbalanced dataset yields the worst unfair results since this is the highest SPD between female and males’ group with LR as 0.38 and 0.43, respectively. Overall, among all DSTs, ADASYN, and SMOTE tend to provide more unfair outcomes compared to other DSTs. In contradiction, they both provide a better classification performance in comparison to RUS and ROS. There is not a huge difference among the three different data unbalanced levels. However, in this study, we experimented with the gender attribute as a sensitive variable.

### Opportunities and challenges

Due to the nature of the CCP process and the rarity issue, training datasets have high chances to have compounded bias and suffer from unbalanced problems not only for the target class but also in the other attributes including sensitive variables. We have noticed that one variable remained unbalanced even after applying the DSTs; in such a case, a careful selection of data attributes should be done to avoid selection bias.

As the quality of training data is important, we would suggest enhanced mechanisms of data repairing techniques to prevent bias in the training data. Furthermore, the algorithmic fairness problem is mostly concerns societal discrimination. For example, in the scholarship selection process, if classifiers give more favors to males than females who have the same qualifications as males but are not selected, this will decrease their chances of scholarship. In a profit-centered industry like telecom, one could think there will be no loss for the customers though any group is less or more favored. It is important to consider the impact of biased decisions for the sake of the company’s reputation, the importance of equal treatment to customers, and to practice ethical AI policies.

## Conclusions

In this paper, we experimented on three versions of unbalanced real-world telecom datasets to assess the impact of using four types of DSTs on the algorithmic fairness in the CCP process and compared the results with the unsampled original dataset. Classification performance and algorithmic fairness were evaluated with well-known metrics. The outcomes imply that RF provides the best classification results. Using SMOTE and ADASYN yields larger SPD between male and female groups as well as a disparate impact on the female over the male group. Previous work emphasizes the use of this method in choosing a scholarship candidate, releasing prisoners on parole, and choosing a credit candidate. Since machine learning applications would be applied to almost every sector in the near future, the practice of using fairer or unbiased systems is essential. Our study highlights the importance of paying attention to algorithmic fairness in the machine-driven decision-making process of the profit-centered and customer-oriented sectors on which very little research work has been done. Particularly, our finding highlights the fact that a careful choice of DSTs must be done to achieve unbiased prediction results. In future work, we would like to test the same procedure on a larger dataset and would like to measure more algorithmic fairness metrics to investigate the best suitable algorithmic measures for the CCP task. Moreover, we would like to test more sensitive variables rather than just gender.

## Data availability

### Underlying data

The real-world telecom dataset was obtained from the Business Intelligence and Analytics department of Telekom Malaysia Bhd. The authors were required to go through a strict approval process following established data governance framework. Interested readers/reviewers may contact the Business Intelligence and Analytics department to request the data ( technicalsuport@tm.com.my). The decision as to whether or not to grant access to the data is at the discretion of Telekom Malaysia Bhd.

As most telco companies own similar customer data, other customer churn datasets that are representative of the data being used in this research can be found as follows:

1. https://www.ibm.com/docs/en/cognos-analytics/11.1.0?topic=samples-telco-customer-churn.
2. https://datasetsearch.research.google.com/search?query=Telco%20Customer%20Churn%20dataset%20site%3Akaggle.com&docid=L2cvMTFsbDF0dzJ5NA%3D%3D.

### Extended data

Analysis code available from: https://github.com/mawmaw/fairness_churn.

Archived analysis code as at time of publication: https://doi.org/10.5281/zenodo.5516218. ^29^

License: MIT License.
