# Supplementary material for: Utilizing data sampling techniques on algorithmic fairness for customer churn prediction with data imbalance problems
Source: F1000Res. 2022 Jun 27;10:988. Originally published 2021 Sep 30. [Version 2] doi: 10.12688/f1000research.72929.2 (PMC9428497; doi:10.12688/f1000research.72929.2)
Supplement: Peer review history

# Peer review: Reviewer response for version 2

**DOI:** 10.5256/f1000research.134711.r148204

**Published:** 2022-08-30

**Authors:** Kok-Chin Khor

**Affiliations:** 1 Department of Internet Engineering and Computer Science, Lee Kong Chian Faculty of Engineering and Science, Universiti Tunku Abdul Rahman, Selangor, Malaysia

DSTs (one under-sampling and three over-samplings) were applied to the three datasets. However, it would be clear for the readers if the authors could specify how many percent of the major class was under-sampled and how many percent of the minor class was over-sampled.

 It is observed that a variable, "is-senior", remained unbalanced even after applying the DSTs. Please explain whether the DSTs (under-sampling or over-sampling?) were applied to the whole dataset dimension or some specific variables only.

 It is stated that the under-sampling technique modifies the data by decreasing the majority of instances, which makes the dataset lack useful information for learning. But this should benefit the classification algorithm as the impact from the major class reduces. The authors can probably explain from the perspective of the rarity of the churn class.

 For the tables, please state the reason certain numbers are bolded. Suggest to put the reason in the caption.

Is the work clearly and accurately presented and does it cite the current literature?

Yes

If applicable, is the statistical analysis and its interpretation appropriate?

Yes

Are all the source data underlying the results available to ensure full reproducibility?

Partly

Is the study design appropriate and is the work technically sound?

Yes

Are the conclusions drawn adequately supported by the results?

Yes

Are sufficient details of methods and analysis provided to allow replication by others?

Partly

Reviewer Expertise:

Data Mining and Computer Networks

I confirm that I have read this submission and believe that I have an appropriate level of expertise to confirm that it is of an acceptable scientific standard.

# Peer review: Reviewer response for version 1

**DOI:** 10.5256/f1000research.76542.r120732

**Published:** 2022-02-24

**Authors:** Prabu P

**Affiliations:** 1 Department of Computer Science, CHRIST University, Bengaluru, Karnataka, 560029, India

This work mainly focus on data imbalance problems in customer churn prediction. The author has to incorporate the following suggestion in his/her article in-order to improve the quality of the work

1. The author need to clearly defined performance metrics for comparing random forest classifier with other classifiers. 
2. More experimental studies required for unequal balancing datasets and author expected to try the experiment with large datasets.
3. Author need to identify some proper technique to manage outliers in the datasets instead of removing manually.
4. Results and Algorithms fairness need to be explain properly.
5. Introduction section are too short and the section is not continuous. Correct it. The introduction section is not clearly explaining the basics and also lack of some details of about "issues in existing work, scope of work, goal of work and organization of work" can be included.
6. No significant limitations are discussed. A number of limitations and learning points are also be considered after the conclusion.
7. Author could take more recent research papers for the literature review and most of the literature review papers are not suitable to the proposed work. Author is expected to include few existing recommender system suggested for e-commerce platform. So I would suggest the author should identify few more recent papers based on his proposed research work and also include the objective and limitations of each work in table format.

Is the work clearly and accurately presented and does it cite the current literature?

Yes

If applicable, is the statistical analysis and its interpretation appropriate?

Partly

Are all the source data underlying the results available to ensure full reproducibility?

Partly

Is the study design appropriate and is the work technically sound?

Yes

Are the conclusions drawn adequately supported by the results?

Yes

Are sufficient details of methods and analysis provided to allow replication by others?

Yes

Reviewer Expertise:

Machine Learning, Cloud Computing ad Mathematical Modeling

I confirm that I have read this submission and believe that I have an appropriate level of expertise to confirm that it is of an acceptable scientific standard, however I have significant reservations, as outlined above.

# Response

**Published:** 2022-06-06

**Authors:** Haw Su Cheng .

1. AUC-ROC score was applied to compare the performance of the respective classifiers and this information is added for clarification in the discussion section/ performance measure section
2. Due to the dataset availability on propriety company, the focus on this paper is on 100,000 records only. Testing with large dataset is put as future work. In future work, we would like to test the same procedure on a larger dataset and would like to measure more algorithmic fairness metrics to investigate the best suitable algorithmic measures for the CCP task. 
3. Since there were not a lot of outliers in the original dataset, they were manually removed and we added this fact in the data pre-processing section.
4. In our study, we do not go further process of mitigating algorithmic unfairness yet. If so, we will need to apply and discuss. But here, we only evaluate and compare the bias in the training dataset before and after applying the data sampling techniques. 
5. Introduction section is updated with related works, scope of work and organization work. Since my background is the combination of three different topics, algorithmic fairness, customer churn prediction and data sampling techniques, Due to the page limitation, could not put a lot of materials in the introduction section but most important and relevant background study was provided in the introduction section in a compact way. Goal of work is provided in the last paragraph of the introduction section, “The main goal of this study is to explore and identify the impact of using DSTs on training data on algorithmic fairness in the CCP process.”
6. We updated with the limitations in the conclusion section as per suggestion.
7. Although there is very limited work which are related to my work, we organize some of the articles of fairness in recommender system in the table format as per your suggestion

# Peer review: Reviewer response for version 1

**DOI:** 10.5256/f1000research.76542.r96164

**Published:** 2022-01-11

**Authors:** Chu Kiong Loo

**Affiliations:** 1 Faculty of Computer Science and Information Technology, University of Malaya, Kuala Lumpur, Malaysia

- This paper addresses an important issue of algorithmic fairness, i.e. to investigate whether the results pose any discrimination between male and female groups and compare the results before and after using DSTs. 
- I suggest to add some discussions on confusion matrix, learning curve and the improvement of fairness index before and after implementing the DSTs.

Is the work clearly and accurately presented and does it cite the current literature?

Yes

If applicable, is the statistical analysis and its interpretation appropriate?

Yes

Are all the source data underlying the results available to ensure full reproducibility?

Yes

Is the study design appropriate and is the work technically sound?

Yes

Are the conclusions drawn adequately supported by the results?

Yes

Are sufficient details of methods and analysis provided to allow replication by others?

Yes

Reviewer Expertise:

Machine learning

I confirm that I have read this submission and believe that I have an appropriate level of expertise to confirm that it is of an acceptable scientific standard, however I have significant reservations, as outlined above.

# Response

**Published:** 2022-06-06

**Authors:** Haw Su Cheng .

Learning curves for Random Forest before and after applying DSTs (for three versions of datasets) are provided. We discussed briefly in the discussion section as well.
